# 3D Printing in Alginic Acid Bath of In-Situ Crosslinked Collagen Composite Scaffolds

**DOI:** 10.3390/ma14216720

**Published:** 2021-11-08

**Authors:** Priscila Melo, Giorgia Montalbano, Sonia Fiorilli, Chiara Vitale-Brovarone

**Affiliations:** 1 Department of Applied Science and Technology, Politecnico di Torino, 10129 Torino, Italy; priscila.soares@polito.it (P.M.); giorgia.montalbano@polito.it (G.M.); chiara.vitalebrovarone@polito.it (C.V.-B.); 2 School of Engineering, Newcastle University, Newcastle upon Tyne NE1 7RU, UK

**Keywords:** in-situ crosslinking, collagen-based systems, 3D printing, alginic acid, mesoporous bioactive glasses, nanohydroxyapatite

## Abstract

Bone-tissue regeneration is a growing field, where nanostructured-bioactive materials are designed to replicate the natural properties of the target tissue, and then are processed with technologies such as 3D printing, into constructs that mimic its natural architecture. Type I bovine collagen formulations, containing functional nanoparticles (enriched with therapeutic ions or biomolecules) or nanohydroxyapatite, are considered highly promising, and can be printed using support baths. These baths ensure an accurate deposition of the material, nonetheless their full removal post-printing can be difficult, in addition to undesired reactions with the crosslinking agents often used to improve the final structural integrity of the scaffolds. Such issues lead to partial collapse of the printed constructs and loss of geometrical definition. To overcome these limitations, this work presents a new alternative approach, which consists of adding a suitable concentration of crosslinking agent to the printing formulations to promote the in-situ crosslinking of the constructs prior to the removal of the support bath. To this aim, genipin, chosen as crosslinking agent, was added (0.1 wt.%) to collagen-based biomaterial inks (containing either 38 wt.% mesoporous bioactive glasses or 65 wt.% nanohydroxyapatite), to trigger the crosslinking of collagen and improve the stability of the 3D printed scaffolds in the post-processing step. Moreover, to support the material deposition, a 15 wt.% alginic acid solution was used as a bath, which proved to sustain the printed structures and was also easily removable, allowing for the stable processing of high-resolution geometries.

## 1. Introduction

Growing innovation in healthcare has propelled the development of new devices and therapies however, problems associated with aging are a constant clinical and economic burden [1]. The existing clinical approaches have provided satisfactory results but, aside from bone grafts, most strategies fail to mimic the natural properties of bone tissue and its composition. Pursuing the solution to this issue has led to the development of new research areas such as bone-tissue engineering (BTE), which aim at creating biomimetic devices that are able to support bone growth and induce its regeneration. To achieve this aim, bioactive materials are created and processed into 3D structures (scaffolds) that actively interact with the surrounding tissues to induce a response that fits the application [2]. Since type I collagen and hydroxyapatite (HA) are the main components of bone tissue, they are often combined into composite materials [3,4] to induce an osteogenic response upon implantation. The appropriate combination between material composition and scaffolds morphology (e.g., porosity and surface properties) can lead to cell growth and proliferation, and possibly to differentiation. To achieve a desired architecture, these scaffolds can be produced using a myriad of techniques, most recently by additive manufacturing (AM), also known as 3D printing [5]. AM allows the design of constructs tuned at the macro and micro-scale, which improves their structural biomimicry while guiding bone regeneration [1,5].

Extrusion is a commonly used printing technique in BTE applications, not only for its versatility regarding the range of materials that can be used, but also for its easy scalability. Despite being a technique that is easily optimized, its use to process collagen-based materials is often accompanied by two main issues: the inherent weak mechanical properties of printed structures and the low viscosity of the collagen-based formulations. These issues propelled the development of new ad hoc strategies able to ensure the stability of the printed strands and an accurate material deposition [6,7].

In previous studies, the authors have created new bioactive biomaterial inks for extrusion printing, consisting of collagen-based suspensions, containing either strontium enriched mesoporous bioactive glasses (MBG_Sr) or nanohydroxyapatite (nanoHA) [7,8]. These inorganic phases are often chosen for their known osteoinductive and osteoconductive abilities, derived from their composition and bioactive properties [9,10]. In details, the high specific surface area of MBGs has been often exploited to realize delivery platforms for biomolecules and therapeutic elements such as strontium that proved to enhance the osteogenic character of the final materials [10]. These collagen-based hybrid formulations were successfully processed into scaffolds, combining extrusion printing with the freeform reverse embedding of suspended hydrogels approach (FRESH), which implies the use of gelatin slurries to support the material printing [11]. After promoting the sol-gel transition of collagen and the simultaneous melting of gelatin upon incubation at 37 °C, the bath was removed and the 3D printed constructs crosslinked using a genipin-based solution. This approach led to collagen-based scaffolds with enhanced stiffness, improved thermal and mechanical stability, and preserved biocompatibility. However, the poor mechanical properties of the constructs before performing the crosslinking step often led to the partial collapse of the structure after the removal of the supporting bath, as well as the loss of scaffold definition. Inspired by the studies of Meng et al. [12] and Paxton et al. [13], this work moves beyond the state-of-the-art exploring a combination of strategies to attain an innovative in-situ crosslinking methodology, to increase the stability of the deposited materials and final printing fidelity. In contrast to most studies regarding in-situ crosslinking of material inks, which involve photochemical reactions (use of photo initiator molecules) [14,15] or the presence of external stimuli, the proposed strategy exploits the combination of the collagen self-assembly capacity and the simultaneous inclusion of a chemical crosslinker into a hybrid printing formulation.

**Figure 1:**
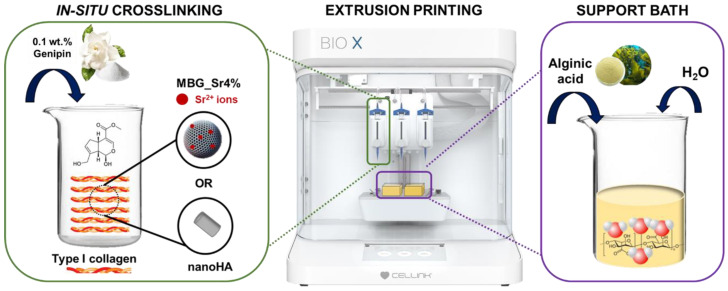
Schematic representation of the developed methodology.

In this study, the authors successfully created in-situ crosslinkable materials, by adding genipin to a preformed collagen formulation enriched with MBG_Sr or nanoHA, where the crosslinker concentration was optimized within a non-cytotoxic range (lower than 0.5 wt.%) (Figure 1) [7,16]. A comprehensive rheological study enabled to investigate the visco-elastic properties of the developed biomaterial formulations, evidencing the effective chemical crosslinking and the consequent gradual stiffening of the composite materials, monitored up to 24 h. Since genipin is known to react with the free amine groups of gelatin to create covalent bonds, an alternative support bath was developed using an alginic acid solution. The newly designed bath proved to be stable and easily removable after printing and incubation at 37 °C. To the best of authors’ knowledge, the presented strategy has not been previously reported and allowed the printing of collagenous systems containing genipin, into scaffolds with different geometries and high resolutions.

## 2. Materials and Methods

### 2.1. Materials

The alginic acid sodium salt was obtained from Sigma Aldrich (Merck KGaA, Darmstadt, Germany). The collagen type I, from bovine Achilles’ tendon was purchased from Blafar Ltd. (Dublin, Ireland) and the genipin powders from Challenge Bioproducts (Touliu, Taiwan). The suspensions were prepared according to the protocols developed by the authors in previous works [8,9] therefore will not be reported in this section, instead were added to the Supplementary Material, alongside the protocol on how to create 1.5 wt.% collagen solutions. The synthesis procedures for strontium-containing MBG nanoparticles (MBG_Sr4%) and rod-like nanosized hydroxyapatite (nanoHA), have been reported by the authors in previous publications and are also resumed in the Supplementary Material, together with the morphological (Figure S1) and structural characterization data (Table S1) [8,10]. The suspension made of type I collagen (1.5 wt.%) and MBG_Sr4% will be reported hereafter as Coll/MBG_Sr4% [9,17], while the suspension containing type I collagen (1.5 wt.%) and rod-like nanoHA will be known as Coll/nanoHA [8]. 

### 2.2. Preparation of Genipin-Containing Collagenous Suspensions: GEN-Coll, GEN-Coll/MBG_Sr4% and GEN-Coll/nanoHA

The protocol described below was used for the preparation of the collagen solutions with and without dispersed nanoparticles (MBG_Sr4% or nanoHA). Genipin-containing collagen solutions will be known as GEN-Coll, and its analog suspensions will be referred as GEN-Coll/MBG_Sr4% and GEN-Coll/nanoHA.

The collagen solution and collagen-based suspensions were prepared, in accordance with the protocol reported by Montalbano et al. [7,8] and stored at 4 °C until use. Two different concentrations of genipin were explored for rheological analysis, which allowed to monitor the variation of the visco-elastic properties of the resulting formulations, alongside the associated crosslinking kinetics. In detail, two homogeneous GEN-Coll solutions were prepared by adding genipin at a concentration of 0.1 wt.% and 0.2 wt.%. The solutions were stirred for 10 min at 4 °C, followed by centrifugation at 2000 rpm (5 min at 10 °C). 

For the printing tests with in-situ crosslinking, the genipin was added to the collagen-based suspensions using the optimized concentration. After stirring for 10 min at 4 °C the resulting formulations were transferred into a 3 mL printing cartridge and centrifuged at 2000 rpm, for 5 min, at 10 °C. 

### 2.3. Preparation of the Alginic Acid Support Bath

The support bath was created by dissolving the alginic acid sodium salt in double distilled water (ddH_2_O) at room temperature, reaching a concentration of 15 wt.%. The solution was left to stir for 3 h, and then transferred into the printing wells for scaffold processing. 

### 2.4. Rheological Testing 

Both suspensions and support bath were characterized for their rheological properties using a DHR-2 controlled stress rotational rheometer (TA Instruments, Waters^TM^, New Castle, Germany) equipped with a parallel plate geometry with a diameter of 20 mm, and a Peltier plate system to constantly control the system’s temperature. The data collected with this instrument was analyzed with the software TRIOS, provided by the rheometer’s manufacturer (TA instruments, Water^TM^, New Castle, Germany).

For the support bath, three types of tests were performed, flow ramp, time sweep and peak hold, setting a temperature of 23 °C. The flow ramp tests (*n* = 3) were done to investigate the variation in the material viscosity over a wide range of shear rates (0.01–1000 s^−1^). The peak hold test studied the changes in viscosity with time through the use of a constant shear rate of 0.001 s^−1^, which was applied for 1 h. Similarly, a time sweep test of 1 h was performed to monitor the visco-elastic properties of the supporting material, setting a constant value for strain (1%) and frequency (1 Hz). Using the same stress conditions, a temperature ramp was applied, ranging from 23 to 37 °C, at a heating rate of 1 °C/min, to investigate the thermal stability of the supporting material.

Both GEN-Coll/MBG_Sr4% and GEN-Coll/nanoHA systems were characterized before and after the sol-gel transition at 37 °C. The suspension’s viscosity was studied with a flow ramp test (*n* = 3), at 10 °C, over a broad range of shear rates (0.01–1000 s^−1^) in order to confirm the material printability. The stability of the material during the printing process (at 10 °C) and its sol-gel transition (at 37 °C) were studied via time sweep tests, where samples were subjected to a fixed strain (1%) and frequency (1 Hz). The duration of the analysis was 2 h for the test at 10 °C, and 1 h for the one at 37 °C. The GEN-Coll solutions containing 0.1 wt.% and 0.2 wt.% genipin were analyzed only by means of oscillatory time sweep tests (1% strain, 1 Hz) at a constant temperature of 10 °C, to explore the potential variation of their visco-elastic properties and define the crosslinking kinetics.

The visco-elastic properties of the systems, after the sol-gel transition at physiological temperature [7,9], were investigated by means of an amplitude sweep test. The analysis was performed on bulk samples created by pipetting 600 µL of suspension containing genipin into a silicone mold (d = 20 mm). The samples were incubated at 37 °C for 3 h and 24 h, prior to analysis. The test was executed by applying an increasing range of strains (0.01–10%), at constant frequency (1 Hz), at 37 °C. Subsequently, a temperature ramp was done within the range of 25–90 °C, using a heating rate of 5 °C/min. This analysis aimed to detect the denaturation temperature of the systems upon crosslinking. 

### 2.5. Strontium Ion Release

The ion release kinetics from the GEN-Coll/MBG_Sr4% samples was investigated by measuring the presence of Sr^2+^ in parts per million (ppm). Bulk samples (*n* = 3) were created by pipetting 400 µL of GEN-Coll/MBG_Sr4% suspension into a silicone mold. Samples were incubated for 24 h, then immersed in 3 mL of Tris HCl buffer (0.1 M, pH 7.4) up to 7 days. The defined time points were 3 h, 10 h, 24 h, 3 days and 7 days. At each time point the media was fully refreshed and the collected supernatant diluted in water at 1:5, to be analyzed by Inductive Coupled Plasma Atomic Emission Spectroscopy (ICP-AES) (ICAP Q, Thermo Scientific, Waltham, MA, USA).

### 2.6. 3D Printing of Collagen Composite Scaffolds

The printability of the materials was investigated using a commercially available 3D Bioprinter (BIOX, Cellink, Gothenburg, Sweden). The materials were printed using a thermally controlled printhead in which the temperature was kept at 10 °C. The printing bed was maintained at room temperature for all tests. All printing experiments were performed using a 3 mL cartridge and a 27 G needle (internal diameter of 0.21 mm). To define the best process conditions, the materials were processed using a variation of process parameters, i.e., different pressures (30–70 kPa), printing speeds (7–10 mm/s) and layer thicknesses (0.17–0.20 mm). The parameters were individually changed to assess their influence in the printing of each suspension. Two different patterns (e.g., grid and honeycomb) were selected to realize the scaffolds, always setting 15% as the infill density. Due to the high number of conducted trials, only the optimized selection of parameters for each material, and geometry, will be reported in the Results section. Post-printing processing consisted of sample incubation at 37 °C for 24 h, followed by the bath removal with a pipette, followed by double washing with ddH_2_O.

The scaffolds measurements after printing were taken with the ImageJ software (National Institute of Health, Bethesda, MD, USA), using the photographs obtained from each selected trial. Measurements were collected for the geometry sides, internal pores, and individual strands. For the determination of the mean pore diameter and strand width, five pores and five strands were chosen for each scaffold (Figure S3), and the final value presented as mean and standard deviation. 

### 2.7. Field Emission Scanning Electron Microscopy (FE-SEM)

Prior to analysis with FE-SEM, samples were frozen at −20 °C for 24 h, and then lyophilized with a Lyovapor L-200 freeze-dryer (Büchi, Cornaredo, Italy) under vacuum (<0.1 mbar) for 24 h. The scaffolds were coated with a 7 nm platinum layer and analyzed with a ZEISS MERLIN instrument (Carl Zeiss AG, Oberkochen, Germany).

### 2.8. Statistics

The results collected from the rheological tests were compared using two-sample t-test, at a significance level of 0.05, using the Welch correction due to high sample variance. The data analysis and plotting were performed with the software OriginPro2016 (Origin Lab Corporation, Northampton, MA, USA). 

## 3. Results

### 3.1. Printability of In-Situ Crosslinking Collagen Hybrid Formulations

**Figure 2:**
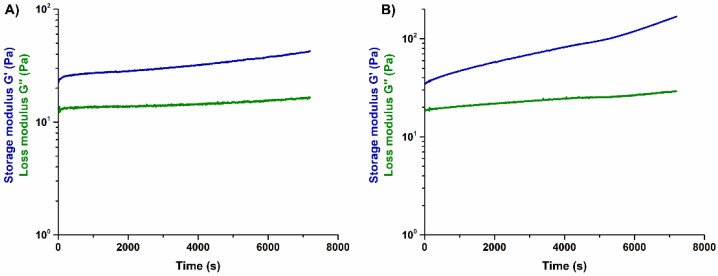
Visco-elastic properties of GEN-Coll solutions containing 0.1 wt.% (**A**) and 0.2 wt.% (**B**) of genipin, at 10 °C.

A concentration of 0.2 wt.% of genipin was initially explored for the in-situ crosslinking of the collagen-based formulations. This concentration was selected from previous studies reporting an effective chemical crosslinking combined with high cytocompatibility of the final material [18]. After the addition of genipin into the collagen solution, rheological analyses were done to investigate the variation of the visco-elastic properties of the material, namely the storage modulus (G′) and the loss modulus (G″). The effect of genipin as a crosslinker was evaluated via time sweep test, which was performed at 10 °C (Figure 2), for 2 h. 

As visible in Figure 2B the solution containing 0.2 wt.% genipin presented an evident and gradual increase of G′, reaching values higher than 100 Pa after 2 h. This was attributed to the effective initial chemical crosslinking of collagen that, in practice, would consequently reduce the time available for printing. 

The concentration of genipin was thus decreased to 0.1 wt.% with the aim to lower the crosslinking reaction rate of the material, thus widening the time interval for the material processing. Accordingly, as shown in Figure 2A, the solution containing 0.1 wt.% genipin displayed constant values of G′ and G″ up to 2 h.

Based on the presented results, a concentration of 0.1 wt.% of genipin was selected in order to guarantee constant visco-elastic properties of the material ink for suitable printing times. This value was used to create the in-situ crosslinking collagen-based suspensions containing either MBG_Sr4% or nanoHA. These hybrid formulations were comprehensively characterized by means of rheological studies to explore their suitability as stable biomaterial inks. 

**Figure 3:**
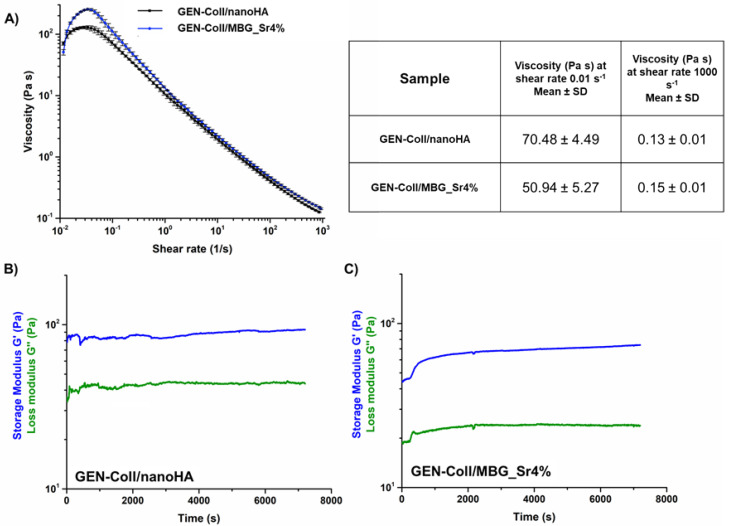
Variation of the viscosity (**A**), and visco-elastic properties of GEN-Coll/nanoHA (**B**) and GEN-Coll/MBG_Sr4%, (**C**) at 10 °C.

Initially, the viscosity of the resulting systems, GEN-Coll/MBG_Sr4% and GEN-Coll/nanoHA, was measured using flow ramp tests, performed at 10 °C. The flow ramp results (Figure 3A) reported a shear-thinning behavior for both suspensions, with viscosity decreasing significantly with increasing shear rates. Following the analysis of the viscosity, time sweep tests were executed to investigate the stability of the suspension at 10 °C. The time sweep tests performed on GEN-Coll/nanoHA and GEN-Coll/MBG_Sr4% (Figure 3B,C) did not show any significant variation of G′ and G″ values up to 2 h, proving the stability of both suspensions at 10 °C while being stored in the printing cartridge.

**Figure 4:**
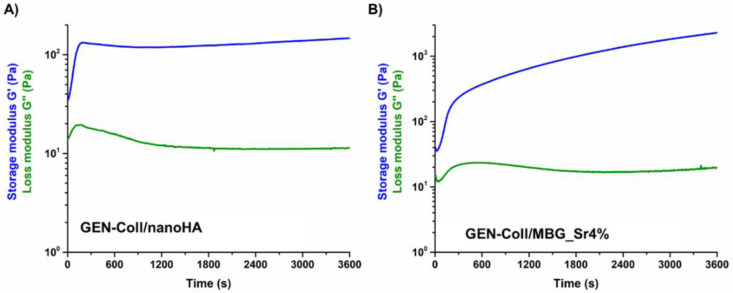
Sol–gel transition of GEN-Coll/nanoHA (**A**) and GEN-Coll/MBG_Sr4% (**B**) at 37 °C.

A time sweep test performed at 37 °C (Figure 4) reported an increase of the visco-elastic properties of both systems, characteristic of the sol-gel transition of collagen at physiological temperature. In addition, higher values of G′ and G″ were registered for the suspension containing MBG_Sr4% as visible in Figure 4B (Table S2).

### 3.2. Effects of Genipin Crosslinking on the Visco-Elastic and Thermal Properties of the Systems upon Reconstitution at 37 °C

**Figure 5:**
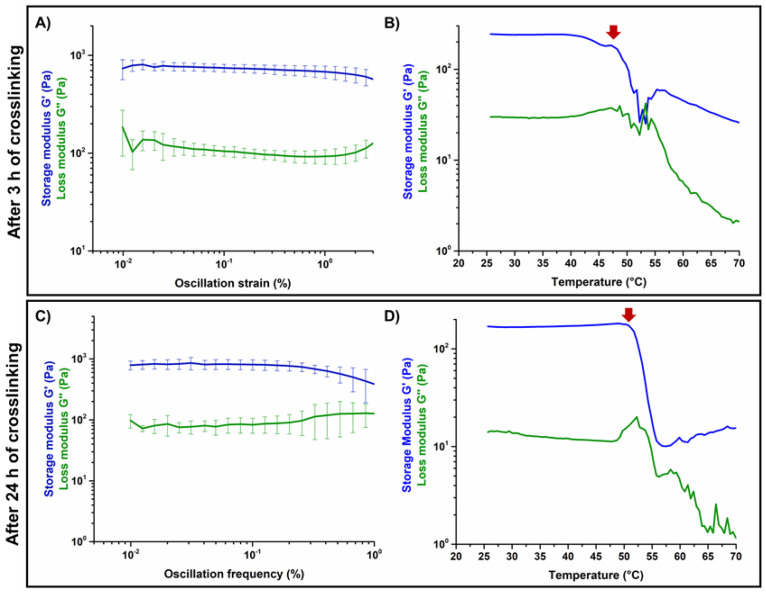
Amplitude sweep test (**A**,**C**) and temperature ramps (**B**,**D**) performed on GEN-Coll/nanoHA after 3 h and 24 h of incubation at 37 °C.

**Figure 6:**
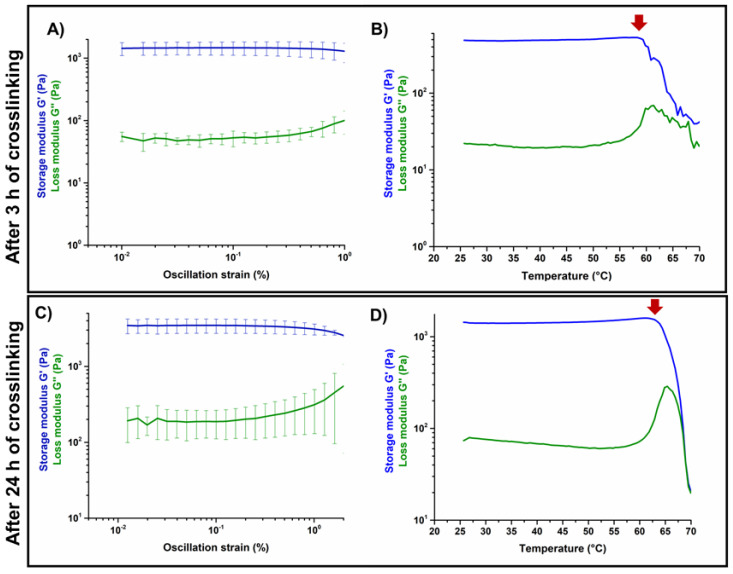
Amplitude sweep test (**A**,**C**) and temperature ramps (**B**,**D**) performed on GEN-Coll/MBG_Sr4% after 3 h and 24 h.

The results of the amplitude sweep tests and temperature ramps performed on GEN-Coll/nanoHA (Figure 5) and GEN-Coll/MBG_Sr4% (Figure 6) inspected the material stiffness and thermal stability after 3 and 24 h of incubation at 37 °C. These tests aimed to study the effectiveness of the in-situ crosslinking, induced by the presence of genipin in the material formulation. For the GEN-Coll/nanoHA system (Figure 5A,C), no significant differences were seen in the visco-elastic properties up to 24 h of incubation at 37 °C. In fact, the values registered for the G′ and G″ were approximately 760 Pa and 140 Pa, respectively (Table S3). A slight difference in the denaturation temperature of the system was observed (Figure 5B,D), demonstrated by a drop of the visco-elastic properties at 47 °C and 52 °C for the samples incubated 3 h and 24 h, respectively. 

Unlike the results observed for the GEN-Coll/nanoHA, which showed little change of G′, the tests realized on the GEN-Coll/MBG_Sr4%, both after 3 and 24 h incubation at 37 °C, demonstrated an increased stiffness and higher thermal stability for this system (Figure 6A,C). This data indicates that an effective crosslinking occurred, shown by a substantial increase of G′ also after 3 h of incubation, suggesting a further extent of crosslinking reaction with consequent stiffer samples by 24 h. Indeed, between 3 h and 24 h the values of G′ (Table S3) went from approximately 1437 Pa (3 h) to 3454 Pa (24 h) (Figure 6A,C). An increment in the denaturation temperature was observed (Figure 6B,D), demonstrating a higher stability of the system compared to the GEN-Coll/nanoHA. After 3 h incubation the decrease of G’ initiated at 59 °C while after 24 h incubation it was observed at 65 °C.

In general, the GEN-Coll/MBG_Sr4% system reported superior visco-elastic properties compared to the GEN-Coll/nanoHA system, even for the shortest period of incubation tested (3 h). The viscoelastic properties of both systems improved after 24 h of incubation, but the GEN-Coll/MBG_Sr4% once more reported higher values for G′. This observation was reflected on the system’s denaturation temperature, which was approximately 12 °C higher for GEN-Coll/MBG_Sr4%, compared to the GEN-Coll/nanoHA, regardless of the incubation time.

### 3.3. Strontium Release of the MBG_Sr4% Containing Formulations

**Figure 7:**
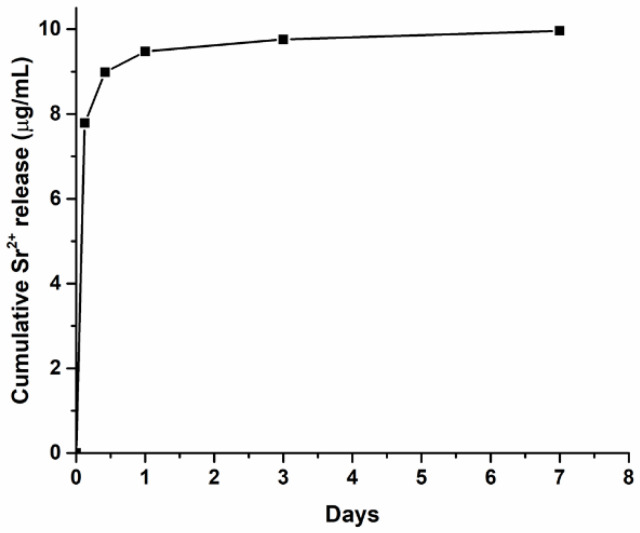
Ion release profile obtained for GEN-Coll/MBG_Sr4% sample throughout 7 days of incubation, measured at specific time points.

The ability of the MBG_Sr4% containing system to release strontium ions was confirmed by means of ICP analysis. In detail, the Sr^2+^ release profile from the GEN-Coll/MBG_Sr4% samples (Figure 7) was similar to the one obtained in previous studies where the material was tested without addition of genipin [17]. In addition, the release of strontium content mostly occurred within the first 3 h of incubation, with no significant leaching observed after 24 h. This shows that the addition of genipin as a crosslinking agent did not hinder the release of Sr^2+^ ions from the final collagen-based formulations. 

### 3.4. Printability of the Hybrid Formulations in Aqueous Alginic Acid Bath (15 wt.%)

**Figure 8:**
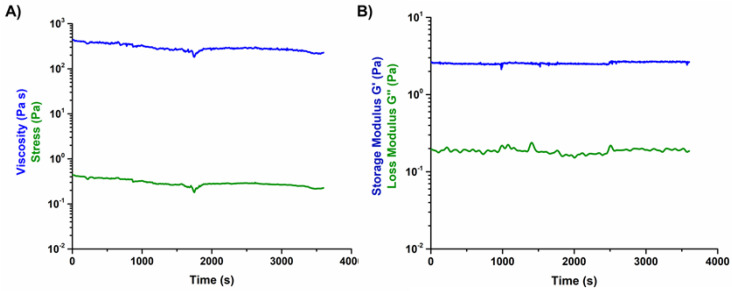
Variation of viscosity and visco-elastic properties of 15 wt.% alginic acid bath with time, at 23 °C, (**A**) viscosity and (**B**) viscoelastic modulus.

Prior to the printing process, a rheological characterization was performed on the developed 15 wt.% alginic acid bath to assess its stability and suitability as support material. The results demonstrated that the material is stable in time, at 23 °C (Figure 8) presenting a constant viscosity (Figure 8A) and G′ after 2 h (Figure 8B). 

**Figure 9:**
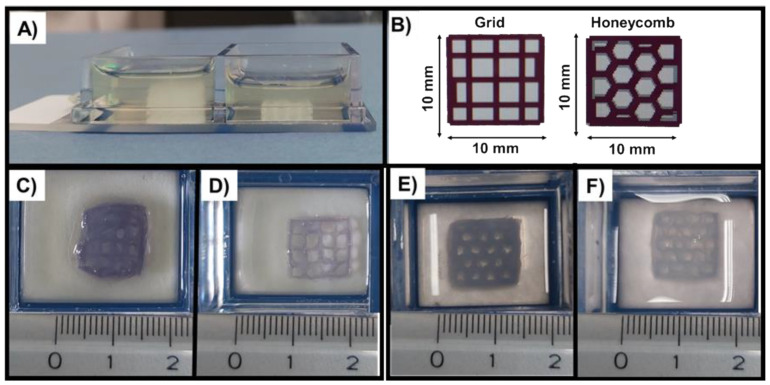
Images of 15 wt.% Alginic acid bath (**A**), scaffold design (**B**) and printed scaffolds after incubation at 37 °C for 24 h: GEN-Coll/MBG_Sr4%, T1 and T2 (**C**,**D**, respectively) and GEN-Coll/nanoHA, T3 and T4 (**E**,**F**, respectively). Blue coloration of scaffolds is indicative of genipin crosslinking. Scaffold measurement 10 × 10 × 1 mm.

To assess the printability of the materials in the 15 wt.% alginic acid bath (Figure 9A), two geometries were chosen (Figure S2): grid (Figure 9C,D) and honeycomb (Figure 9E,F). The scaffolds were printed using the parameters listed in Table 1, then measured with ImageJ (Table S4), alongside the resultant CAD provided by the BIOX printer (Table S5). 

As previously mentioned, several trials were performed, changing each parameter individually, to ensure a good control of the process and a maximum understanding of the results. However, only the best conditions are reported. The changes performed were based on the effects of each parameter on the printing fidelity and final scaffold resolution. To avoid continuous needle clogging, the smallest needle usable was 27 G, which conditioned the minimum layer height to 0.17 and 0.18 mm. According to the literature, the layer height is normally set as 60–70% of the internal needle diameter, in order to improve the final scaffold resolution and to control the amount of material deposited [13]. In parallel, increasing the pressure leads to a higher quantity of material being extruded, a factor that can be controlled by tuning the printing speed, which is associated with the amount of material deposited in each point. Based on these considerations, a fine-tuning of the mentioned parameters is needed as they are strictly intertwined. 

A visual analysis of the scaffolds produced (Figure 9C–F) immediately shows that the geometries were successfully replicated, with all structures presenting defined pores and strands. A more in-depth assessment was done with ImageJ, allowing the comparison between the measurements of the printed scaffolds with those of the 3D CAD models. 

Starting with the GEN-Coll/MBG_Sr4% system, using the parameters defined in T1 (Figure 9C), the resulting scaffold geometry was similar to that of the CAD file, as demonstrated by the measurements taken to the sides of the scaffold, where values are comparable to those desired (within the 10 mm range) (Table 2). Scaffolds produced at a pressure of 50 kPa, 8 mm/s printhead speed and 0.18 mm layer height (T1) showed a pore diameter that was approximately 330 µm smaller, and strands 2 times wider, than those of the original model. This clearly points to an excessive deposition of material, which could be solved via two routes: (1) reducing the extrusion pressure, (2) decreasing the layer height. Reducing the pressure in previous trials led to insufficient deposition of material, even at lower printing speeds, which is associated with the material viscosity that imposes a minimum threshold on the pressure needed to push the material out of the needle. Based on this, the layer height was decreased to the minimum allowed by the system, 0.17 mm (T2), resulting in a significant improvement of the printing resolution, also represented in the final measurements, which matched those of the BIOX file.

The trials performed with GEN-Coll/nanoHA system started using the settings of T2 as these provided the best results for the GEN-Coll/MBG_Sr4% suspension, yet the outcome was not satisfactory. The resultant honeycomb scaffolds (T3) (Figure 9E) appeared bulky, with visual absence of some of the pores, ascribable to an excess of material deposition. Moreover, the strands obtained were nearly twice as wide compared to the CAD file confirming that an excessive amount of material was deposited. To decrease the amount of material extruded, the pressure was lowered to 40 kPa, as for this material, it did not present problems in previous trials. This change improved the printing resolution however, the high speed did not allow for the material to settle. A decrease in the printing speed to 7 mm/s immediately improved the accuracy of the material deposition, which translated into higher shape fidelity. Nonetheless, the amount of material was slightly low, and some areas appeared to lack it. This was solved by increasing the layer height to 0.18 mm, resulting in improved shape fidelity (T4), confirmed by the closer values obtained for the measured pore diameter and strand width. Using the optimized parameters both formulations were printed in the form of grid and honeycomb (Figure S2).

### 3.5. Morphological Assessment of the Resulting 3D Scaffolds

**Figure 10:**
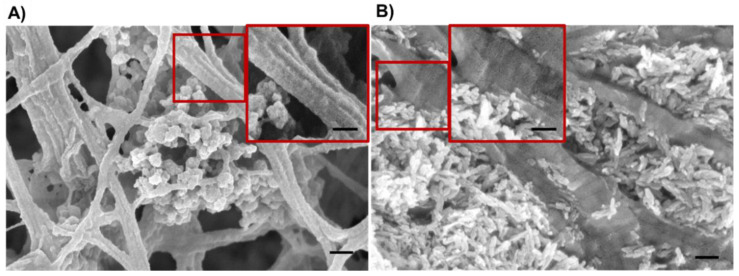
FE-SEM micrographs of produced scaffolds after freeze-drying. (**A**) GEN-Coll/MBG_Sr4% and (**B**) GEN-Coll/nanoHA. Collagen D bands seen in both samples and zoomed in on area delimited by the red squares. Scale bar 100 nm.

The morphology of the scaffolds was subsequently analyzed via FE-SEM after lyophilization (Figure 10), which reported the successful distribution of the nanoparticles within the collagenous matrix (Figure S7), evidencing the nanoparticles homogeneous embedding. Collagen D bands were observed, as expected from previous studies on similar systems [7,8,9]. 

## 4. Discussion

### 4.1. Influence of Genipin Content on the Rheological Behavior of the Hybrid Formulations

The processability of collagen-based suspensions via extrusion printing can be challenging due to the soft nature of the material and its incapability to sustain itself once deposited. This issue was studied in a previous investigation performed by the authors, using similar suspensions [7,11]. In the mentioned studies, mesh-like scaffolds were successfully printed inside a gelatin support bath, using the method known as the freeform embedding of suspended hydrogels (FRESH). Despite the positive results regarding the geometrical resolution of the scaffolds, after printing the constructs were not stable which led to their partial collapse [11]. In attempt to stabilize the structure during printing, and prior to the removal of the support bath, an in-situ crosslinking methodology was combined with the material’s ability to self-assemble, leading to the results presented in this study.

Genipin was chosen to attain the in-situ crosslinking of the materials and, as a first test, it was initially added to a base collagen solution (1.5 wt.%) to study the optimal amount needed to obtain a printable material that was stable for at least 2 h. Based on the rheological characterization of the collagen solutions containing either 0.1 wt.% and 0.2 wt.% of genipin, the concentration selected was 0.1 wt.% as it provided a longer printing time without alterations to the materials’ viscosity. Moreover, according to the literature, this concentration of genipin is not cytotoxic [16].

Once the amount of crosslinker was optimized, the visco-elastic properties of the obtained formulations were characterized at the established printing temperature (10 °C), in order to assess their suitability as extrudable material inks. As demonstrated by the time sweep analysis, both suspensions proved to be stable at 10 °C up to 2 h after the addition of genipin (Figure 2). This result was considered particularly promising since an early material jellification would cause the variation of its visco-elastic properties as well as potential nozzle blockage, hindering the entire printing process [13,19]. At variance, the assessed stability can allow flexibility of the process and provides a wider processing time, prior to the material’s chemical crosslinking, and subsequent sol-gel transition.

Considering that the collagen self-assembly occurs at 37 °C, the suspensions containing either MBG_Sr4% or nanoHA were incubated at this temperature to promote the reconstitution of a solid matrix [20,21]. The material was then subjected to a time sweep analysis to explore the effective material transition. The data indicated that both materials presented a transition from sol to gel, with the suspension Coll-MBG_Sr4% presenting a higher increase in G′, compared to the Coll-/nanoHA (Figure 3). The rapid increase of G′ indicates the formation of collagen fibrils due to the physical crosslinking of collagen molecules and the resultant reconstitution of a solid matrix [7,8,21]. The lower degree of crosslinking, demonstrated by the G′ values of the system containing nanoHA, could be associated with the interactions between HA and collagen. Briefly, according to Kim et al. [22], genipin acts by crosslinking collagen free amino groups, through the formation of cyclic structures which act as intra- and inter-molecular bridges along the fibers. In collagen-HA composites, two types of main interactions can occur: electrostatic interactions, between opposite charged components, and/or hydrogen bonding [23,24]. The electrostatic interactions are particularly strong between exposed Ca^2+^ cations on HA surface and the carboxylate groups of collagen. Using Fourier Transform Infrared analysis (FTIR), Chen et al. observed a small shift in the absorption peaks of the amide I, when comparing pure collagen to a collagen-HA composite [25]. These observations may explain the lower interactions between genipin and collagen molecules compared to the Coll/MBG_Sr4% system, also resulting in a less effective crosslinking.

To investigate the effects of genipin in the visco-elastic properties and thermal denaturation of crosslinked samples, two time points were chosen: 3 h and 24 h (after the addition of genipin). The nanoHA-containing systems showed similar visco-elastic properties after 3 and 24 h of incubation, meaning that the crosslinking time did not affect their mechanical performance. Concerning the denaturation temperature, an increase of 7 °C was observed when samples were crosslinked for 24 h however, these values are still within the range of non-crosslinked collagen and thus the increase is not significant [26]. Better results were obtained for the Coll-MBG_Sr4%, regardless of the crosslinking time, where an evident increase in the material visco-elastic properties, as well as denaturation temperature, was observed after 24 h of incubation, compared to 3 h. The statistics performed between sample types, revealed these differences were significant, indicating that this crosslinking strategy is more effective for the Coll/MBG_Sr4% system.

Finally, the ion release profile of the samples containing Sr^2+^ (GEN-Coll/MBG_Sr4%) was not affected by the addition of genipin, confirming the results of previous work by the authors [17]. This is a positive outcome as the incorporation of this ion is key to improve both osteoconductivity and osteoinductivity of the developed systems [9,10,27].

### 4.2. Suitability of the Alginic Bath for the Printing Process

The approach developed in this study is based on the 3D printing of soft materials using a sacrificial support bath. In the literature, these baths are known as suspension media, and provide a platform for the deposition of mechanically weak materials (e.g., low viscosity suspensions), into complex, well-defined structures [6]. Unlike common extrusion printing processes, where material is deposited on a flat surface in air, this approach implies their deposition into a bath that sustains the printed material, avoiding the collapse of the final structure. In one of their studies, the authors used the FRESH method, where a gelatin slurry served as the support bath. In this work, once optimized the genipin content into the material formulation (0.1 wt.%), preliminary printing trials were done to assess if the crosslinker would react with the support bath, the aforementioned gelatin. The GEN-Coll solution was thus printed into a gelatin bath and incubated at 37 °C for 24 h. As expected, the results demonstrated that the genipin added to the solution also crosslinked the support bath material surrounding the scaffold (Figure S4), causing the partial loss of geometrical accuracy. In this work, to overcome this drawback, an alternative bath that would not react with genipin was explored, based on an aqueous solution of 15 wt.% alginic acid. During the printing process, the bath is kept at room temperature, therefore its visco-elastic properties were assessed at 23 °C. In agreement with observations by other authors [27,28], the alginic bath showed a shear thinning behavior (Figure S5), which is associated with the material’s molecular structure and organization. When under shear stress, the polysaccharide chains slither through one another, leading to their disengagement, known as the reptation process. The abrupt decrease observed at a low shear rate (1 s^−1^) has been previously seen for low concentration alginate gels [29].

Considering the potential diffusion of genipin into the supporting material after printing, a flow ramp test was performed on alginic acid containing 0.1 wt.% genipin (Figure S5). The results showed that there was no significant difference between viscosity values obtained with and without genipin, ensuring that the chosen crosslinker does not interact with the supporting material. Since the printing process may vary in duration depending on the scaffold architecture and size, the stability of the support bath was tested over time, using a peak hold flow test, where the shear rate was kept constant. A low shear rate value was used to mimic the quasi-static conditions of the bath during printing. The acquired data reported that the viscosity remains constant for 1 h, as well as the stress applied, suggesting no significant material changes during the process. The visco-elastic properties are also important, as these can change in time and with temperature, especially for polymeric solutions [27]. In the presented protocol, samples are printed at room temperature, and then incubated at 37 °C to promote, and accelerate, the crosslinking reaction. The results obtained from Figure 8B ensure the bath stability at 23 °C for 1 h, through a constant value of G′. The G″ curve is slightly noisy which is associated with a heterogeneity within the material structure, where parts of the material are stiffer due to higher molecular interaction. This heterogenous character is also seen when the sample was subjected to changes in temperature. For temperature higher than 33 °C (Figure S6), an abrupt decrease is seen for G′, associated with a higher level of disentanglement of the molecular chains. Application-wise, this could be ideal as it will facilitate the bath removal during the post-processing stage.

### 4.3. Printability of the In-Situ Crosslinking Suspensions in the Alginic Acid Bath (15 wt.%) and Post-Processing

Extrusion-based printing is a known technique in the area of regenerative medicine, and highly applied to the processing of hydrogels [30]. The used technique has an accuracy range in the order of 1 µm, which is a major challenge for the production of scaffolds with high shaping fidelity, especially for soft materials such as collagen, which are not able to self-sustain [6,30].

The printed scaffolds presented a high geometrical definition, similar to that of the original CAD files (Table 2), and the produced geometries possessed a high shape fidelity, therefore overcoming the aforementioned challenge [30]. To assess the printability of the materials, several factors must be considered, including the material properties and application, the process parameters, and the components of the system (e.g., nozzle diameter). In this context, all these factors were considered in order to optimize the printing of the developed suspensions. The materials were characterized using rheological testing which allowed the understanding of their behavior in time, at different shear stresses and temperatures. Then, the principal parameters of the process were gradually tested, to achieve the optimal protocol for each system. The geometry of the constructs was not a target of the study, being the two models chosen as proof of concept, assumed as having an equal degree of complexity.

During printing the materials behaved differently, hence the parameters deemed ideal for the GEN-Coll/MBG_Sr4% (T2) were inadequate when applied to the GEN-Coll/nanoHA suspension (T3), resulting in excessive deposition of material, thus a final bulky structure. For GEN-Coll/nanoHA, a lower pressure and printing speed appeared to be best, allied to the increase in layer height (T4). The overall dimension of the scaffolds, defined as 10 × 10 mm, was also met as shown by the values recorded in Table 2.

Pore diameter and strand width are also key parameters in defining the printability of a material [5,31]. Despite the signs of material dragging due to the movement of the bath material, using the optimized conditions, the measurements of these features matched those of the designed scaffold, implying a high printing resolution. This confirms that the alginic acid bath developed not only supported the material, but also allowed for its accurate deposition.

The post-processing was easily carried out, without experiencing difficulties, contrary to what is often reported in the literature. In most studies concerning the use of a support bath, two common problems are mentioned: (1) the extraction of the construct from its embedment in the support bath, which requires the use of elevated temperatures, changing of the pH conditions or even the use of enzymatic digestion; (2) the disparity between the temperature of the ink and the support bath, which might induce drastic changes to the physical and chemical states of the bath itself, or affect the printed material [6]. In this study, the low viscosity of the alginic acid solution, allowed for its easy removal at 23 °C and 37 °C, by simply pipetting out the material, thus reducing the risk of damaging the scaffolds’ integrity. Moreover, considering the low concentration of the solution, a simple washing with ddH_2_O allowed the removal of any residual material that might be trapped in the pores of the printed scaffold. In addition, the in-situ crosslinking enhanced the mechanical stability of the scaffolds, further reducing the risk of structure damage and collapse. Subsequent improvement of the process could consist on setting a temperature of 23 °C to promote a preliminary stabilization of the construct, favoring the natural process of collagen self-assembly [20,21].

## 5. Conclusions

In conclusion, the visco-elastic properties of collagen-based hybrid formulations containing strontium enriched MBGs or HA nanoparticles were enhanced by the addition of 0.1 wt.% genipin, allowing for in-situ crosslinking of the material. A clear difference was seen between the two printed composite constructs, with the MBG-containing formulation (GEN-Coll/MBG_Sr4%) showing higher stiffness values, as well as a superior denaturation temperature. The lower performance of GEN-Coll/nanoHA composite was attributed to the larger extent of established interactions between HA particles and collagen fibers, which led to a lower amount of available amino groups, therefore hindering the chemical crosslinking. This indicates that, for this strategy, MBG-containing systems should be preferred. Additionally, their ability to deliver therapeutic Sr^2+^ ions was confirmed: a desired feature in devices targeting bone regeneration. Despite the difference in the final visco-elastic properties, both formulations were stable during the printing process, allowing for long printing time intervals, thus suitable for the manufacturing of complex geometries.

The use of an alginic acid bath proved to be an added asset to the developed methodology, being a valid alternative to the gelatin bath used in the FRESH method. Being a water-based supporting material, easily removable by washing with aqueous solutions, it represents a sustainable approach, potentially translatable at industrial level.

Finally, both biomaterial inks were successfully processed into scaffolds with high printing fidelity, using basic patterns (grid and honeycomb) and needles with small diameter, confirming the potential of the developed in-situ crosslinking methodology.

Altogether, these results contributed to the improvement of the printing fidelity of soft materials and facilitated the handling and post-processing of the resulting scaffolds, which can be further crosslinked to enhance their visco-elastic properties and increase their denaturation temperature.

## Figures and Tables

**Table 1:** Best printing parameters used to process the different in-situ crosslinking suspensions. All trials were performed with a 27 G needle and setting a 15% infill of each layer.

Trial	Composition	Geometry	Speed (mm/s)	Pressure (kPa)	Layer Thickness (mm)
T1	GEN-Coll/MBG_Sr4%	Grid	8	50	0.18
T2			8	50	0.17
T3	GEN-Coll/nanoHA	Honeycomb	8	50	0.17
T4			7	40	0.18

**Table 2:** Measurements obtained for the scaffolds and original BIOX CAD derived from ImageJ measurements presented Figure 9. Information regarding GEN-Coll/MBG_Sr4%, T1 and T2 (Figure 9C,D, respectively) and GEN-Coll/nanoHA, T3 and T4 (Figure 9E,F, respectively).

Samples	Sides (mm)				Pores (mm) Mean ± SD	Strand (mm) Mean ± SD
Original grid	9.44	10.16	9.47	10.03	1.89 ± 0.36	0.56 ± 0.07
Grid (Figure 9C)	11.58	10.65	10.41	10.31	1.33 ± 0.15	1.19 ± 0.12
Grid (Figure 9D)	10.17	10.44	10.35	10.26	1.91 ± 0.11	0.62 ± 0.14
Original honeycomb	9.52	9.95	9.44	10	1.86 ± 0.26	0.72 ± 0.19
Honeycomb (Figure 9E)	12.01	12.14	11.65	11.61	1.33 ± 0.17	1.38 ± 0.15
Honeycomb (Figure 9D)	11.25	10.89	12.14	11.43	1.30 ± 0.15	0.82 ± 0.13

## Data Availability

Not applicable.

## Supplementary Materials

The Supplementary Material for this article provides additional information to the reader on protocols and data, allowing for a deeper insight into the study performed. The following are available online at https://www.mdpi.com/article/10.3390/ma14216720/s1. Figure S1. FESEM analysis of nanoparticles used to create the hybrid formulations. (A) MBG_Sr4% and (B) rod-like nanoHA. Table S1. Surface characterization of MBG_Sr4% obtained from [2] and size, including for nanoHA from Montalbano et al. Figure S2. 3D printing of grid and honeycomb geometries using (A,B) GEN-Coll/MBG_Sr4% and (C,D) GEN-Coll/nanoHA. Figure S3. Measurement points used for the ImageJ analysis. (A) T1; (B) T2; (C) T3; (D) T4. Table S2. Visco-elastic properties of collagen-based suspensions at 37 °C, obtained from the time sweep tests (1 h). Values registered after 6 s and 3600 s. Table S3. Values obtained for G′ and G″ at different strains, obtained from the amplitude sweep tests, performed on GEN-Coll/nanoHA and GEN-Coll/MBG_Sr4%, after 3 h and 24 h of incubation at 37 °C. Table S4. Set of measurements obtained from ImageJ analyses for the produced scaffolds. Table S5. Set of measurements obtained from ImageJ analysis for the original CAD files obtained from the BIOX. Figure S4. Scaffolds printed in a gelatin bath support, made with suspensions containing 0.1 wt.% genipin. Figure S5. Variation of the viscosity of the 15 wt.% alginic acid bath, with and without 0.1 wt.% genipin. Figure S6. Temperature ramp performed on 15 wt.% alginic acid bath.
