# Supplementary material for: 3D Printing in Alginic Acid Bath of In-Situ Crosslinked Collagen Composite Scaffolds
Source: Materials (Basel). 2021 Nov 8;14(21):6720. doi: 10.3390/ma14216720 (PMC8588345; doi:10.3390/ma14216720)
Supplement: Supplementary file 1 [file materials-14-06720-s001.zip › materials-1418171-supplementary.pdf]

materials

Supplementary Information

3D Printing in Alginic Acid Bath of In-Situ Crosslinked Collagen Composite Scaffolds

Priscila Melo [1,2], Giorgia Montalbano [1], Sonia Fiorilli [1]* and Chiara Vitale-Brovarone [1]

[1] Department of Applied Science and Technology, Politecnico di Torino, 10129 Torino, Italy; priscila.soares@polito.it (P.M.); giorgia.montalbano@polito.it (G.M.); sonia.fiorilli@polito.it (S.F.); chiara.vitalebrovarone@polito.it (C.V.-B.)

[2] School of Engineering, Newcastle University, NE1 7RU Newcastle Upon Tyne, UK; priscila.melo@newcastle.ac.uk

* Correspondence: sonia.fiorilli@polito.it

Citation: Melo, P.; Montalbano, G.; Fiorilli, S.; Vitale-Brovarone, C. 3D Printing in Alginic Acid Bath of In Situ Crosslinked Collagen Composite Scaffolds. *Materials* **2021**, *14*, 6720. https://doi.org/10.3390/ma14216720

Academic Editor: Giovanni Vozzi

Received: 27 September 2021
Accepted: 2 November 2021
Published: 8 November 2021

Publisher's Note: MDPI stays neutral with regard to jurisdictional claims in published maps and institutional affiliations.

Copyright: © 2021 by the authors. Licensee MDPI, Basel, Switzerland. This article is an open access article distributed under the terms and conditions of the Creative Commons Attribution (CC BY) license (http://creativecommons.org/licenses/by/4.0/).

Protocol for the synthesis of rod-like nano-HA particles adapted from Montalbano et al.[1].

5.48 g of potassium phosphate dibasic trihydrate were dissolved in 100 mL of doubled distilled water. Successively, 0.2 vol% of Darvan 821-A was added to the solution and the pH was set to 10.5 through the addition of 1 M sodium hydroxide solution. In order to have a Ca/P molar ratio of 1.67, a second solution obtained by dissolving 4.44 g of calcium chloride in 60 mL of doubled distilled water was prepared and, after 1 h of stirring, added dropwise into the solution containing the phosphate precursor while constantly maintaining the pH at 10.5 through the addition of sodium hydroxide. The resulting solution was kept under stirring for 3 h, constantly maintaining the pH at 10.5. After an overnight ageing step performed at atmospheric pressure and room temperature, the supernatant was removed, and the remaining slurry was poured in a 250 mL Teflon-lined hydrothermal reactor and placed in an oven at 100 °C for 4 h. After cooling, the supernatant was removed, and the resulting slurry was centrifuged in order to separate HA particles. The latter were washed three times with distilled water and once with pure ethanol. In the end, nano-HA particles were collected in a Petri dish and dried in an oven at 100 °C for 24 h.

Protocol for the synthesis of MBG_Sr4% particles adapted from Fiorilli et al. [2].

MBG nanoparticles containing 4 mol % of Sr, molar ratio Sr/Ca/Si = 4/11/85 (MBG_Sr4%) were synthesised by a base-catalysed sol-gel synthesis. In particular, 6.6 g cetyltrimethylammonium bromide (CTAB ≥98%, Sigma Aldrich, Milan, Italy) and 12 mL NH_4OH (Ammonium hydroxide solution, Sigma Aldrich, Milan, Italy) were dissolved in 600 mL of ddH_2O under stirring for 30 min. Then, 30 mL of tetraethyl orthosilicate (TEOS, Tetraethyl orthosilicate, Sigma Aldrich, Milan, Italy), calcium nitrate tetrahydrate ($Ca(NO_3)_2 \cdot 4H_2O$, 99%, Sigma Aldrich, Milan, Italy) and strontium chloride hexahydrate ($SrCl_2 \cdot 6H_2O$, for analysis EMSURE® ACS), were added and kept under vigorous stirring for 3 h. The powder was collected by centrifugation (Hermle Labortechnik Z326, Hermle LaborTechnik GmbH, Wehingen, Germany) at 10,000 rpm for 5 min, washed once with distilled water and two times with absolute ethanol. The final precipitate was dried at 70 ◦C for 12 h and then calcined at 600 ◦C in air for 5 h at a heating rate of 1 ◦C min^{-1} using a furnace (Carbolite 1300 CWF 15/5 Carbolite Ltd., Hope Valley, UK), in order to remove CTAB.

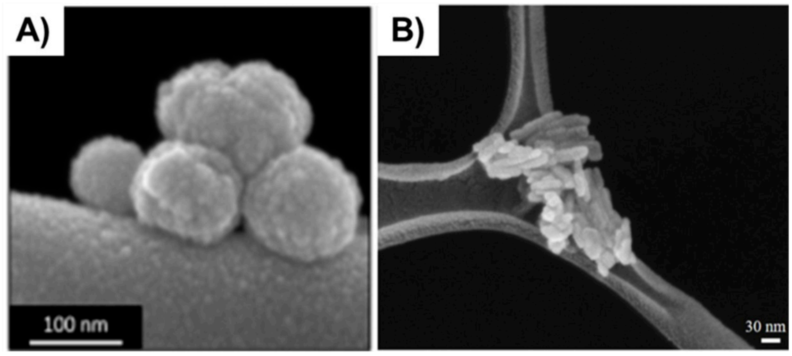

Figure S1. FESEM analysis of nanoparticles used to create the hybrid formulations. (**A**) MBG_Sr4% and (**B**) rod-like nanoHA[1, 2].

Table S1. Surface characterisation of MBG_Sr4% obtained from [2] and size, including for nanoHA from Montalbano et al.[1].

Parameters	MBG_Sr4%	nanoHA
BET surface area ($m^2.g^{-1}$)	551	NA
Average pore size (nm)	4.1	NA
Pore volume ($cm^3.g^{-1}$)	0.45	NA
Particle size (nm)	100-200	20-80

Protocol for the preparation of collagen-based suspensions [1, 3]

The following materials were obtained from Sigma Aldrich: 0.5 M acetic acid and sodium hydroxide (NaOH). Darvan 821-A was provided by Vanderbilt Minerals. The collagen type I, from bovine Achilles' tendon was purchased from Blafar Ltd. (Dublin, Ireland).

The process starts with the preparation the collagen type I solution, where collagen powders were dissolved in 0.5 M acetic acid at a 1.5 wt% concentration, and stirred overnight, at 4 °C. For the suspensions containing MBG (Coll-MBG_Sr4%), the inorganic phase particles were suspended in 0.5 M acetic acid and further sonicated for 60 min in an ultrasonic bath (Digitec DT 103H, Bandelin, Berlin, Germany). The suspension was added to the collagen solution, dropwise, under stirring at 4 °C, and the final suspension neutralized with 1 M NaOH to achieve pH 7.4. For the suspensions containing nanoHA (Coll-nanoHA), the nanoparticles were suspended in a solution containing 1 M NaOH and Darvan 821-A, sonicated for 1 h and left to stir overnight at 4 °C. Finally, the suspension was added to the collagen solution, dropwise, resulting in a neutral suspension (pH 7.4).

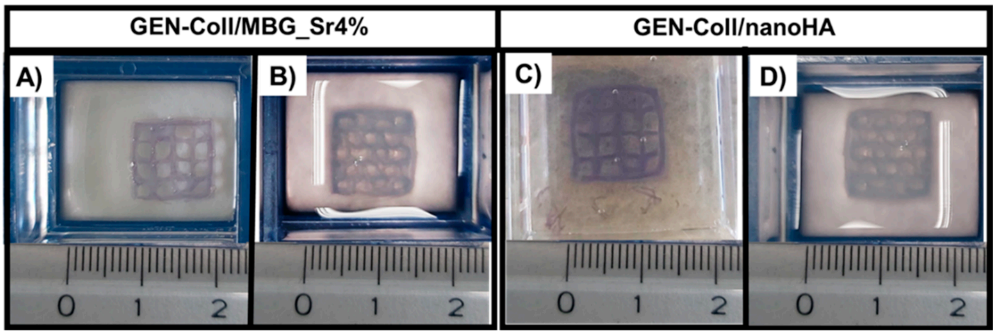

Figure S2. 3D printing of grid and honeycomb geometries using (**A**,**B**) GEN-Coll/MBG_Sr4% and (**C**,**D**) GEN-Coll/nanoHA.

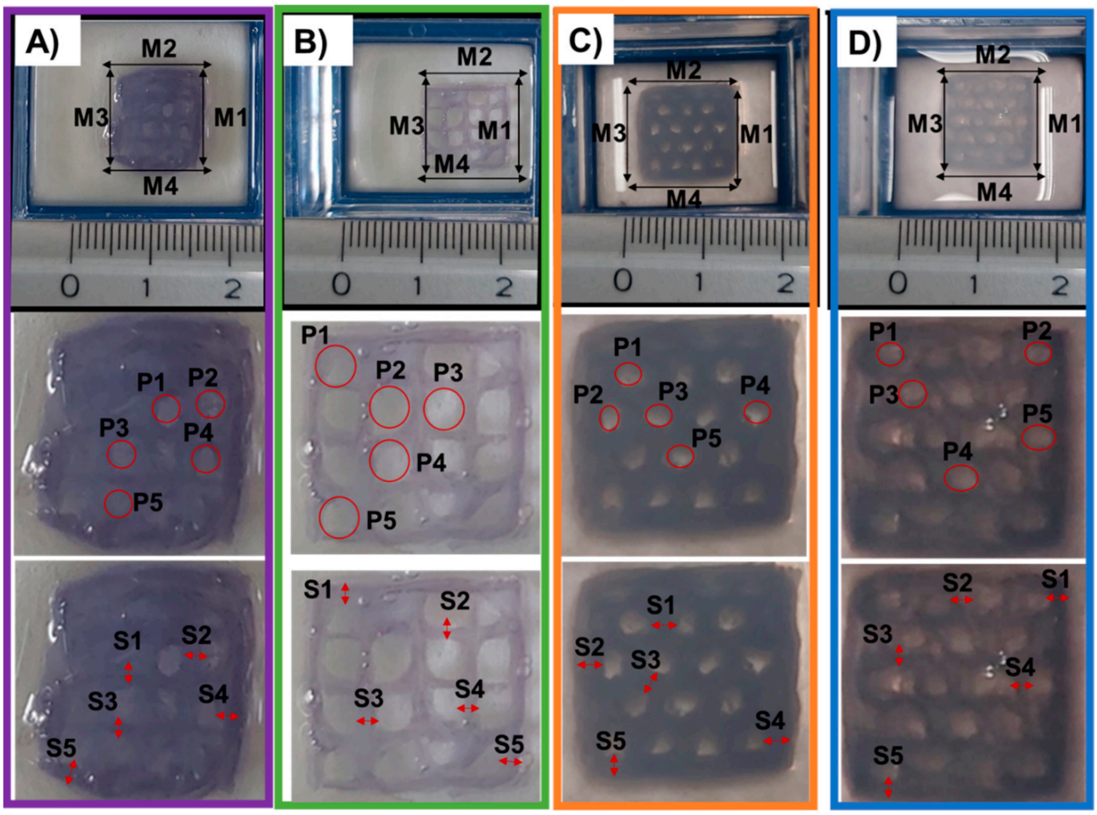

Figure S3. Measurement points used for the ImageJ analysis. (**A**) T1; (**B**) T2; (**C**) T3; (**D**) T4.

Supplementary figures of Section 3.

Table S2. Visco-elastic properties of collagen-based suspensions at 37 °C, obtained from the time sweep tests (1 h). Values registered after 6 s and 3600 s.

Samples	Storage Modulus G′ (Pa)<br>Loss Modulus G″ (Pa)	
	**6 s**	**3600 s**
GEN-Coll/nanoHA	G′: 78.48<br>G″: 36.16	G′: 146.86<br>G″: 11.33
GEN-Coll/MBG_Sr4%	G′: 44.33<br>G″: 18.66	G′: 2284.61<br>G″: 19.76

Table S3. Values obtained for G′ and G″ at different strains, obtained from the amplitude sweep tests, performed on GEN-Coll/nanoHA and GEN-Coll/MBG_Sr4%, after 3 hours and 24 hours of incubation at 37 °C.

Viscoelastic Properties of Bulk Samples		Storage Modulus G′ (Pa)<br>Loss Modulus G″ (Pa)			
		**GEN-Coll/nanoHA**		**GEN-Coll/MBG_Sr4%**	
		**3h**	**24 h**	**3h**	**24h**
Amplitude sweep (37 °C)	No strain	G′: 734.87<br>G″: 184.11	G′: 792.06<br>G″: 97.55	G′: 1437.87<br>G″: 55.49	G′: 3454.88<br>G″: 191.74
	10 % strain	G′: 135.04<br>G″: 198.82	G′: 7.47<br>G″: 35.56	G′: 126.51<br>G″: 250.61	G′: 589.48<br>G″: 590.37

Table S4. Set of measurements obtained from ImageJ analyses for the produced scaffolds. Information regarding GEN-Coll/MBG_Sr4%, T1 and T2 (Figure 9C,D, respectively) and GEN-Coll/nanoHA, T3 and T4 (Figure 9E,F, respectively).

Samples	Point of measure	Grid (Figure 9C)	Grid (Figure 9D)	Honeycomb (Figure 9E)	Honeycomb (Figure 9D)
Sides (mm)	M1	11.58	10.17	12.01	11.25
	M2	10.66	10.44	12.14	10.89
	M3	10.41	10.35	11.65	12.14
	M4	10.31	10.26	11.61	11.43
Pore diameter (mm)	P1	1.52	1.86	1.52	1.10
	P2	1.26	2.09	1.07	1.22
	P3	1.35	1.94	1.52	1.49
	P4	1.09	1.91	1.34	1.46
	P5	1.44	1.77	1.21	1.25
Strand width (mm)	S1	1.31	0.49	1.61	0.80
	S2	1.18	0.49	1.37	0.98
	S3	1	0.84	1.28	0.77
	S4	1.35	0.75	1.16	0.60
	S5	1.16	0.55	1.46	0.92

Table S5. Set of measurements obtained from ImageJ analysis for the original CAD files obtained from the BIOX.

Samples	Point of measure	Grid (Figure 9B)	Honeycomb (Figure 9B)
Sides (mm)	M1	9.44	9.52
	M2	10.16	9.95
	M3	9.47	9.44
	M4	10.03	10
Pore diameter (mm)	P1	1.44	1.97
	P2	2.18	1.52
	P3	2.13	2.19
	P4	2.23	2.05
	P5	1.46	1.60
Strand width (mm)	S1	0.51	0.96
	S2	0.61	0.70
	S3	0.45	0.39
	S4	0.61	0.80
	S5	0.64	0.77

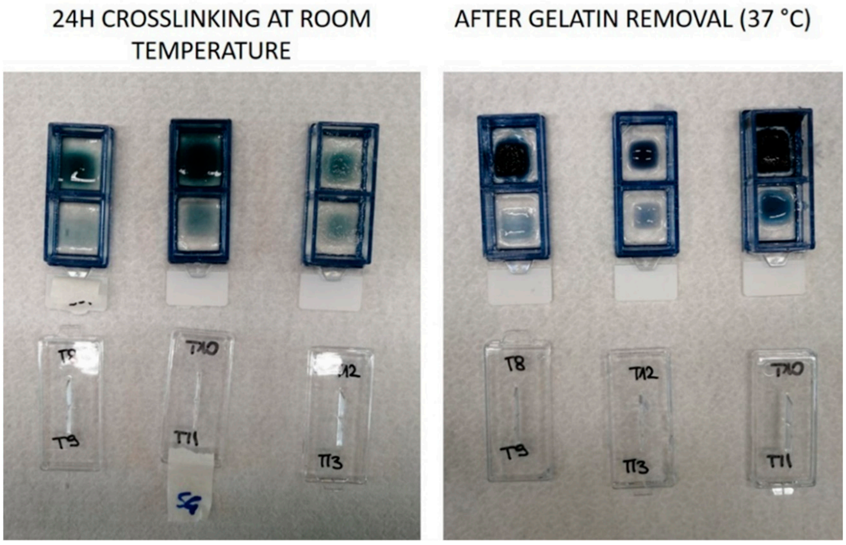

Figure S4. Scaffolds printed in a gelatine bath support, made with suspensions containing 0.1 wt.% genipin.

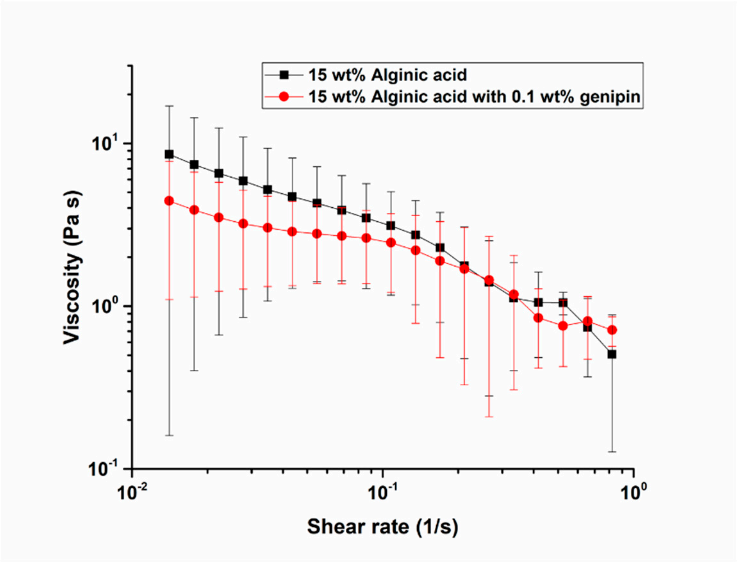

Figure S5. Variation of the viscosity of the 15 wt.% alginic acid bath, with and without 0.1 wt.% genipin.

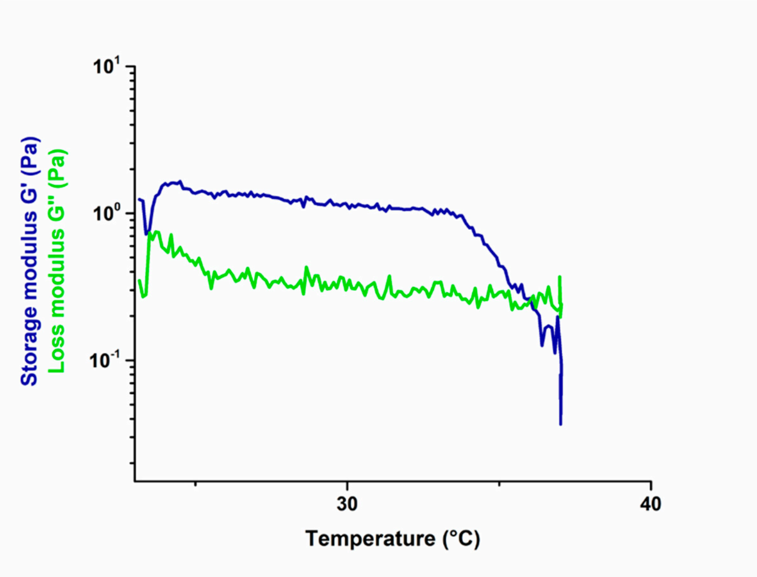

Figure S6. Temperature ramp performed on 15 wt.% alginic acid bath.

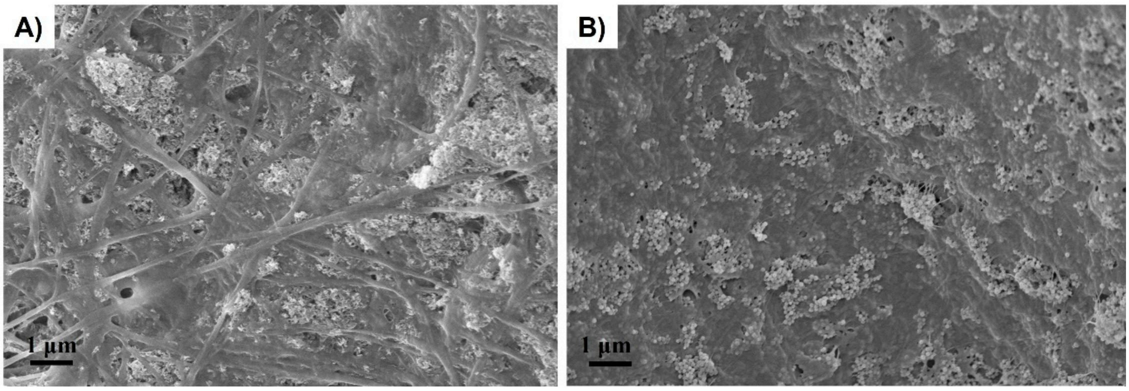

Figure S7. FESEM micrographs of produced scaffolds after *in-situ* crosslinking, showing the homogeneous distribution of the (**A**) nanoHA and (**B**) MBG_Sr4% particles, respectively.

Referenses

1. Montalbano G, Molino G, Fiorilli S, Vitale-Brovarone C (2020) Synthesis and incorporation of rod-like nano-hydroxyapatite into type I collagen matrix: A hybrid formulation for 3D printing of bone scaffolds. J Eur Ceram Soc. https://doi.org/https://doi.org/10.1016/j.jeurceramsoc.2020.02.018
2. Fiorilli S, Molino G, Pontremoli C, et al (2018) The Incorporation of Strontium to Improve Bone-Regeneration Ability of Mesoporous Bioactive Glasses. Mater (Basel, Switzerland) 11:. https://doi.org/10.3390/ma11050678
3. Montalbano G, Borciani G, Cerqueni G, et al (2020) Collagen hybrid formulations for the 3d printing of nanostructured bone scaffolds: An optimized genipin-crosslinking strategy. Nanomaterials 10:1–23. https://doi.org/10.3390/nano10091681
